# Neurocognitive assessments and long-term outcome in an adult with 2-methyl-3-hydroxybutyryl-CoA dehydrogenase deficiency

**DOI:** 10.1016/j.ymgmr.2018.06.005

**Published:** 2018-06-22

**Authors:** Karolina M. Stepien, Philomena McCarthy, Eileen P. Treacy, James J. O'Byrne, Gregory M. Pastores

**Affiliations:** a National Centre for Inherited Metabolic Diseases, The Mater Misericordiae University Hospital, Dublin, Ireland; b University College Dublin, Ireland

**Keywords:** Neurocognitive function, Psychometric test, Cognitive function, Long-term follow- up, MHBDD-2, Methyl-3-hydroxybutyryl-CoA dehydrogenase deficiency, IMD, Inherited Metabolic Diseases, ABAS, Adaptive Behaviour Assessment System, WISC, Wechsler Intelligence Scale for Children, WAIS, Wechsler Adult Intelligence Scale, TONI, The Test of Nonverbal Intelligence, IQ, Intelligence Quotient

## Abstract

**Background:**

2-Methyl-3-hydroxybutyryl-CoA dehydrogenase deficiency (MHBDD) is a rare X-linked disorder associated with the accumulation of 2-methyl-3-hydroxybutyric acid in body fluids as a consequence of a disruption in isoleucine metabolism. The clinical presentation is heterogeneous, including a neurodegenerative course with retinopathy and cardiomyopathy leading to death in early childhood and a slowly progressive disease associated with learning disability and survival into adulthood. The condition is often diagnosed in childhood.

**Results:**

This paper outlines the long-term neurocognitive outcomes in a 38-year old man with MHBDD. Several psychometric tests were used to assess his cognitive ability and adaptive functioning in childhood during an acute illness and in adulthood when the patient showed deterioration in the ability to walk or speak.

**Conclusions:**

There is an increasing demand for an accurate and objective measure of cognitive functioning that can be used to follow the natural progression of MHBDD. Psychological assessment may enable the identification of organic problems. The application and interpretation of psychometric tests used in children may vary from those used in adults.

## 1 Background

2-Methyl-3-hydroxybutyryl-CoA dehydrogenase deficiency (MHBDD) is a rare X-linked disorder associated with the accumulation of 2-methyl-3-hydroxybutyric acid in body fluids due to abnormal activity of MHBDD which is involved in isoleucine degradation [1]. Degradation of the branched-chain amino acid isoleucine in humans takes place in mitochondria via the concerted action of a series of enzymes, during which isoleucine first undergoes transamination to 2-keto-3-methylbutyrate, followed by oxidative decarboxylation to 2-methylbutyryl-CoA [2].

Almost 30 patients with MHBDD have been reported worldwide. To date, seven pathogenic variants in *HADH2* have been reported to result in MHBDD. Among missense pathogenic variants, p.Arg130Cys is the most common [[2], [3], [4], [5]].

The classical infantile form of MHBDD disease is characterized by a progressive neurodegenerative course with retinopathy and cardiomyopathy, leading to death at the age of 2–4 years or later [6]. Clinical presentation, however, can be very heterogeneous [7], ranging from diarrhoea, vomiting, lethargy, disturbance of consciousness, dyspnea and metabolic acidosis during acute illness, to slow neurocognitive decline in adulthood [5, 8]. Some patients may present with developmental delay or neurologic problems without regression and patients without neurologic regression during youth may develop neurologic problems and/or other symptoms later in life. Indeed Lorea et al. (2015) reported a patient who had a *HADH2* p.Ala158Val variant and developed Parkinsonism at the age of 27 years [9].

Most patients with MHBDD described so far presented in childhood; in a few cases diagnosis was not established until adulthood [9, 10]. This paper presents the long-term neurocognitive outcomes in an adult patient affected with MHBDD.

## 2 Results (CASE)

A 23-year old man was biochemically diagnosed with juvenile MHBDD, as described previously by Olpin et al. [10]. Ten years later, sequence analysis of *HADH2* led to identification of a novel, likely deleterious pathogenic variant, c.745G>C (p.Glu249Gln). His mother was an asymptomatic carrier.

**Fig. 1:**
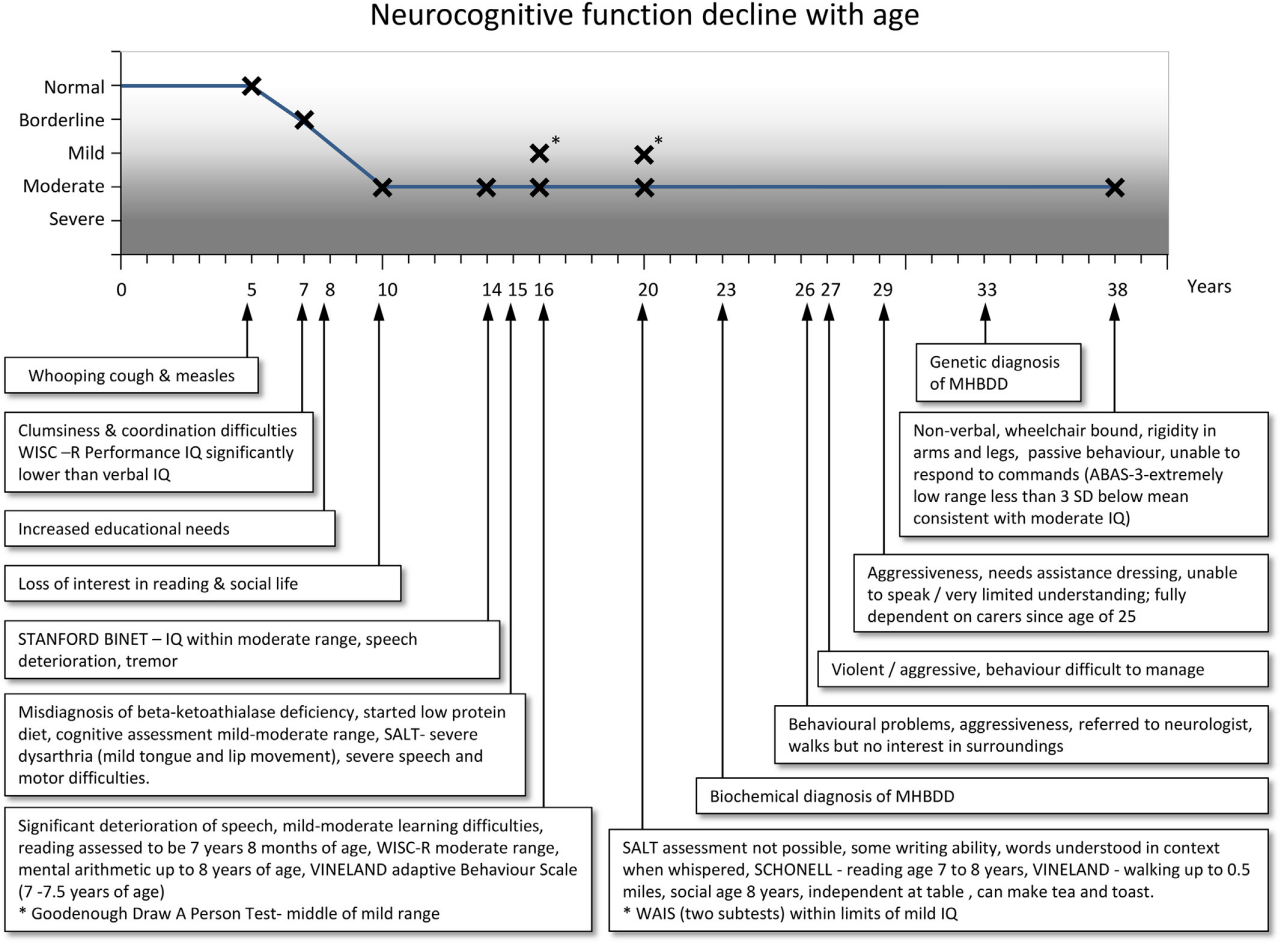
Neurocognitive function decline with age; x axis-age (years), y axis- neurocognitive function; NORMAL- similar to peers of the same age; BORDERLINE- IQ 70-85; MILD-IQ 50 -55 to 70; MODERATE-IQ 35-40 to 50-55; SEVERE- IQ 20 -25 to 35 -40. SALT- Speech and Language Therapy; WISC-R- Wechsler Intelligence Scale for Children; ABAS 3- Adaptive Behaviour Assessment System; WAIS- Wechsler Adult Intelligence Scale.

At the age of 38, he presented to the Adult Metabolic Clinic with significant speech and motor impairment. He displayed a passive behaviour, and although he was unable to verbally communicate, he smiled when engaged. The clinical course of his mild condition and his steady neurocognitive impairment are presented in Fig. 1.

On examination he exhibited tremor and signs of dystonia treated with carbidopa and levodopa (Sinemet 62.5 mg daily). He had marked contractures in his knee and ankle joints and was unable to walk independently. He had marked muscle atrophy (sarcopenia), attributed to lack of mobility and chronic wheelchair use and intake of a low protein diet (0.7 g/kg) since childhood. Audiometry showed he had normal ipsilateral acoustic reflexes indicating healthy VIIIth nerve pathways and hearing was assessed as not worse than mild loss.

His biochemical tests showed plasma ammonia, renal function and liver function tests within normal reference ranges. Plasma amino acids showed leucine 121 μmol/L (38–83), isoleucine 64 μmol/L (77–162) and valine 205 μmol/L (151–302). Acylcarnitine profile was normal and free carnitine was 16.6 μmol/L. His most recent urine organic acids profile showed a mild increased excretion of 2-methyl-3-hydroxybutyrate, a moderate increase in excretion of tiglylglycine with no 2-methylacetoacetate detected. A slight increase in vanillactate and vanilpyruvate acids detected were likely to be secondary to carbidopa and levodopa intake prescribed for tremor and dystonia.

Table 1 outlines psychology assessments that were performed at different stages of his life.

**Table 1:** Psychometric tests used in a patient with 2-methyl-3-hydroxybutyryl-CoA dehydrogenase deficiency over a period of 38 years.

Psychometric test	Used in child/adult	Outcomes measured	Validated for assessment in diseases	References
Adaptive Behaviour Assessment System-3 (ABAS-3)	Used with children and adult populations (0–89 years)	Assessment of adaptive skills needed to independently care for oneself, respond to others and meet the demands at home, school work and in the community.	Norm referenced based on US data	Harrison & Oakland [25]
Goodenough Draw-a- Person Test	Children 2–13 years	A nonverbal measure of intelligence.	Normative data unknown to author	Goodenough-Harris [26]
Schonell Reading Test	Children and adults	Reading ability	A nonverbal measure of intelligence	
Stanford-Binet Intelligence Scale fourth Edition	Children and adults from 2 to 85 years	- Intelligence (IQ) - Fluid reasoning - Knowledge - Quantitative reasoning - Visual-spatial processing - Working memory	Normed on a representative sample of 4800 individuals, children/adults	Thorndike et al. [27]
Vineland Adaptive Behaviour Scales	0–90 years	Measures personal and social skills from birth through to adulthood	- Adults with intellectual disabilities in residential care/non-residential facilities (ambulatory/non-ambulatory) - Children with hearing and vision impairment	Sparrow et al. [28]
Wechsler Intelligence Scale for Children-revised (WISC-R)	6–16 years	- Intelligence - Verbal Comprehension Index - Visual Spatial Index - Fluid Reasoning Index - Working Memory Index - Processing Speed Index	- UK and US norms including - Intellectually gifted children - Children with mild or moderate intellectual disability -Children with specific learning disorders (reading, written expression and math) - Children with ADHD/disruptive behavior - Children who are English Language Learners - Children with autism spectrum disorder with language impairment - Children with autism spectrum disorder without language impairment - Children with traumatic brain injuries - Not normed on metabolic population but valid for those with mild/moderate intellectual disability	Wechsler [29]
	16–90 years (+11 months)			
Wechsler Adult Intelligence Scale				
				Wechsler [30]

The patient completed the Wechsler Intelligence Scale for Children (WISC) at 7 and 16 years of age and the Wechsler Adult Intelligence Scale (WAIS) at age 20 years. His scores on these tests were reported to be within the mild, moderate and mild range respectively. At 7 years, there was statistically significant discrepancy between his Performance Intelligence Quotient (IQ) (mild range) and his Verbal IQ (low average range). The patient completed the Stanford Binet at 10 years indicating an IQ within the Moderate range.

It was not possible to re-administer the WAIS at 38 years due to the patient's limited receptive capacity and significantly limited motor and verbal skills. Information was gathered from clinical interview with his mother, and his adaptive functioning was assessed using the Adaptive Behaviour Assessment System-Third Edition (ABAS-3). The patient's general Adaptive Composite Score on the ABAS-3 was reported to be in the extremely low range (<0.1 percentile). This score is consistent with a moderate IQ in the absence of a comprehensive IQ assessment. His reading was assessed at 16 and 20 years using the Schonell Reading Test, and his level when retested remained stable between 7 and 8 years. His social functioning as assessed on the Vineland Adaptive Behaviour Scale was between 7 and 8 years. This domain was assessed on the ABAS-3 and was 3 standard deviations (SD) below the mean (0.2 percentile). It was reported that from the age of 26 he was fully dependent on carers for daily living skills such as dressing, toileting, and feeding. Reportedly around the same time, the patient began to display challenging and aggressive behaviour.

## 3 Discussion

The natural history of MHBDD disease is not clear in view of a small number of cases described. There are distinct differences in the phenotype of the condition with sensory, cognitive, and behavioural manifestations varying considerably [7]. This case, probably the longest living patient with this rare disease, illustrates the challenges associated with and importance of a regular psychology review to assess cognitive function in a patient with an Inherited Metabolic Disease (IMD).

There is a paucity of data on the long-term outcomes in patients with IMDs and no clear guidelines or consensus on what battery of psychological tests to use in such cases. The Wechsler Intelligence Scales are the gold standard in IQ tests and are not normed on a population of patients with IMD. Monitoring IQ and adaptive functioning in patients with IMD at various time points using appropriate tests may facilitate tracking disease progression and inform care planning, educational/occupational opportunities, interventions and the efficacy of different therapy options.

This patient had several tests performed throughout his life (Fig. 1 and Table 1). Various tests were administered in this case, aimed at assessing different cognitive functions to detect the magnitude of changes, if any, over time (Table 1). The review of his previous assessments revealed that deterioration occurred in the patient's verbal and fine and gross motor skills. In retrospect, it was difficult to determine which tests were best to monitor the patient's cognitive and adaptive function due to the limited findings reported. However, it appears that all tests administered (see Table 1) were helpful at the time in guiding educational/occupational placements and identifying the need for further neurological investigation.

Assessments of IQ were performed using the standardized Wechsler Intelligence Scales. Variations in IQ test results may reflect changes in assessment measures used or differences in administration from one examiner to the next [11]. However, correlations on the Wechsler Scales between current and earlier versions may detect subtle changes [11, 12]. In this patient, assessments were performed by different psychologists over the period of observation, and different subtests were administered which may have impacted on the overall score.

It may be important to consider certain tests that are appropriate for the patient and can be used throughout the lifespan. If tests are to be used longitudinally (e.g., Wechsler Intelligence Scales and Stanford Binet), accurate reporting of results will allow for tracking of IQ throughout the lifespan. Several publications have reported on the use of Wechsler Intelligence Scales in patients with an IMD. Whilst neuropsychologic research into the cognitive development of patients with an IMD has been limited to standardized intelligence tests, achievement tests and adaptive behaviour scales [[13], [14], [15]] are not always suitable. Long-term follow-up of neuropsychological functioning in patients with metabolic disorders remains difficult due to limited opportunities for comprehensive neuropsychological evaluations [16]. Identifying tests that can be administered to those who have significantly limited verbal and motor skills may be relevant in some patients with IMD. For instance, the Test of Nonverbal Intelligence fourth Edition (TONI-4) for those age 6–89 years (+11 months) assesses non-verbal intelligence requiring minimal physical response.

### 3.1 Cognitive assessment during the acute illness

Psychological assessment may call attention to the presence of organic problems, and may help clinicians identify changes in executive function and facilitate early intervention. Early identification and initiation of appropriate treatment may prevent or delay developmental and spasticity problems [17]. Emerging concerns should prompt further specific executive function testing [18].

In our case all psychology assessments were performed at the time of acute illness such as whooping cough/measles, or when learning difficulties were evident and proved to be useful in identifying sudden deterioration in cognitive function or a change in disease course. Specific recommendations were made following early assessment (e.g., appropriate school placement).

### 3.2 Dementia and cognition

Degradation of the branched-chain amino acid isoleucine in humans takes place in the mitochondria via the concerted action of a series of enzymes, including MHBDD [2]. It has been speculated that multifunctional enzymes might play a role in the pathogenesis of Alzheimer's disease [2, 19]. Cognitive assessments are difficult to administer and interpret, when undertaken in individuals with moderate to profound intellectual disability, as they rely on verbal communication, particularly receptive communication [20]. Moreover, respondents often need to use fine motor skills such as writing and drawing, and many individuals with moderate to severe intellectual disability have poor hand dexterity [20, 21]. In addition, most assessments lack sensitivity in terms of differentiating levels of lower intellectual functioning [20], or responses cannot be scored properly [20, 22].

### 3.3 Treatment and neurocognitive assessment

Treatment applied before brain damage occurs generally results in better cognitive outcomes [23]. The efficacy of the applied low protein diet used for MHBDD in the context of neurocognitive function has never been fully evaluated, although it was shown to reduce the number of episodes of acute decompensation [24]. Our patient remained on a low protein diet throughout his life. Despite having a steady and gradual decline in his neurocognitive status, he has never experienced any acute decompensation in his life.

## 4 Conclusions

There is an increasing demand for measures of cognitive function that are able to accurately and objectively track the natural progression of IMDs. Psychological assessment may enable the identification of organic problems. A psychologist is an important member of a multi-disciplinary team in metabolic medicine to be able to carry out assessment when required and to use this information to support the patient's management. Further work is required to validate the psychometric tests on a larger group of adult patients with rare disease.

## Consent for publication

The patient's family gave their full informed consent.

## Ethical approval and consent to participate

N/A

## Availability supporting data

N/A

## Competing interests

KMS, PM, EPT, JOB, GMP have no conflict of interest for this publication.

## Funding

N/A

## Authors' contributions

KMS and PM - conception and design, literature search and drafting the manuscript.

EPT, JOB and GMP -revising the manuscript critically for important intellectual content.

All authors read and approved the manuscript before submission.

## Authors' information

N/A
